# MIBiG 3.0: a community-driven effort to annotate experimentally validated biosynthetic gene clusters

**DOI:** 10.1093/nar/gkac1049

**Published:** 2022-11-18

**Authors:** Barbara R Terlouw, Kai Blin, Jorge C Navarro-Muñoz, Nicole E Avalon, Marc G Chevrette, Susan Egbert, Sanghoon Lee, David Meijer, Michael J J Recchia, Zachary L Reitz, Jeffrey A van Santen, Nelly Selem-Mojica, Thomas Tørring, Liana Zaroubi, Mohammad Alanjary, Gajender Aleti, César Aguilar, Suhad A A Al-Salihi, Hannah E Augustijn, J Abraham Avelar-Rivas, Luis A Avitia-Domínguez, Francisco Barona-Gómez, Jordan Bernaldo-Agüero, Vincent A Bielinski, Friederike Biermann, Thomas J Booth, Victor J Carrion Bravo, Raquel Castelo-Branco, Fernanda O Chagas, Pablo Cruz-Morales, Chao Du, Katherine R Duncan, Athina Gavriilidou, Damien Gayrard, Karina Gutiérrez-García, Kristina Haslinger, Eric J N Helfrich, Justin J J van der Hooft, Afif P Jati, Edward Kalkreuter, Nikolaos Kalyvas, Kyo Bin Kang, Satria Kautsar, Wonyong Kim, Aditya M Kunjapur, Yong-Xin Li, Geng-Min Lin, Catarina Loureiro, Joris J R Louwen, Nico L L Louwen, George Lund, Jonathan Parra, Benjamin Philmus, Bita Pourmohsenin, Lotte J U Pronk, Adriana Rego, Devasahayam Arokia Balaya Rex, Serina Robinson, L Rodrigo Rosas-Becerra, Eve T Roxborough, Michelle A Schorn, Darren J Scobie, Kumar Saurabh Singh, Nika Sokolova, Xiaoyu Tang, Daniel Udwary, Aruna Vigneshwari, Kristiina Vind, Sophie P J M Vromans, Valentin Waschulin, Sam E Williams, Jaclyn M Winter, Thomas E Witte, Huali Xie, Dong Yang, Jingwei Yu, Mitja Zdouc, Zheng Zhong, Jérôme Collemare, Roger G Linington, Tilmann Weber, Marnix H Medema

**Affiliations:** Bioinformatics Group, Wageningen University, Droevendaalsesteeg, 6708 PB Wageningen, The Netherlands; The Novo Nordisk Foundation Center for Biosustainability, Technical University of Denmark, Kgs. Lyngby, Denmark; Bioinformatics Group, Wageningen University, Droevendaalsesteeg, 6708 PB Wageningen, The Netherlands; Westerdijk Fungal Biodiversity Institute, Uppsalalaan 8, 3584 CT Utrecht, The Netherlands; Scripps Institution of Oceanography, University of California San Diego, 9500 Gilman Drive, La Jolla, CA 92093-0212, USA; Department of Microbiology and Cell Science, University of Florida, Gainesville, FL 32611, USA; Department of Chemistry, University of Manitoba, 66 Chancellors Cir, Winnipeg, MB R3T 2N2, Canada; Department of Chemistry, Simon Fraser University, 8888 University Drive, Burnaby, British Columbia V5A 1S6, Canada; Bioinformatics Group, Wageningen University, Droevendaalsesteeg, 6708 PB Wageningen, The Netherlands; Department of Chemistry, Simon Fraser University, 8888 University Drive, Burnaby, British Columbia V5A 1S6, Canada; Bioinformatics Group, Wageningen University, Droevendaalsesteeg, 6708 PB Wageningen, The Netherlands; Department of Chemistry, Simon Fraser University, 8888 University Drive, Burnaby, British Columbia V5A 1S6, Canada; Unnatural Products, 2161 Delaware Ave. Suite A, Santa Cruz, CA 95060, USA; Centro de Ciencias Matemáticas UNAM, Morelia, México; Department of Biological and Chemical Engineering, Aarhus University, Denmark; Department of Chemistry, Simon Fraser University, 8888 University Drive, Burnaby, British Columbia V5A 1S6, Canada; Bioinformatics Group, Wageningen University, Droevendaalsesteeg, 6708 PB Wageningen, The Netherlands; Food and Animal Sciences, Department of Agricultural and Environmental Sciences, Tennessee State University, Nashville, TN 37209, USA; Department of Chemistry, Purdue University, West Lafayette, IN, USA; Department of Applied Sciences, University of Technology, Iraq; Bioinformatics Group, Wageningen University, Droevendaalsesteeg, 6708 PB Wageningen, The Netherlands; Institute of Biology, Leiden University, Sylviusweg 72, 2333BE Leiden, The Netherlands; Laboratorio Nacional de Genómica para la Biodiversidad-Unidad de Genómica Avanzada, Cinvestav. Km 9.6 Libramiento Norte Carretera Irapuato-León, CP 36824 Irapuato, Gto., México; Institute of Biology, Leiden University, Sylviusweg 72, 2333BE Leiden, The Netherlands; Laboratorio Nacional de Genómica para la Biodiversidad-Unidad de Genómica Avanzada, Cinvestav. Km 9.6 Libramiento Norte Carretera Irapuato-León, CP 36824 Irapuato, Gto., México; Institute of Biology, Leiden University, Sylviusweg 72, 2333BE Leiden, The Netherlands; Laboratorio Nacional de Genómica para la Biodiversidad-Unidad de Genómica Avanzada, Cinvestav. Km 9.6 Libramiento Norte Carretera Irapuato-León, CP 36824 Irapuato, Gto., México; Departamento de Microbiología Molecular, Instituto de Biotecnología, Universidad Nacional Autónoma de México, Cuernavaca, Morelos, México; Synthetic Biology and Bioenergy Group, J. Craig Venter Institute, La Jolla, CA 92037, USA; Bioinformatics Group, Wageningen University, Droevendaalsesteeg, 6708 PB Wageningen, The Netherlands; Institute of Molecular Bio Science, Goethe-University Frankfurt, D-60438 Frankfurt am Main, Germany; LOEWE Center for Translational Biodiversity Genomics (TBG), Senckenberganlage 25, 60325 Frankfurt am Main, Germany; The Novo Nordisk Foundation Center for Biosustainability, Technical University of Denmark, Kgs. Lyngby, Denmark; School of Molecular Sciences, University of Western Australia, Perth, Australia; Institute of Biology, Leiden University, Sylviusweg 72, 2333BE Leiden, The Netherlands; Departamento de Microbiología, Instituto de Hortofruticultura Subtropical y Mediterránea ‘La Mayora’, Universidad de Málaga-Consejo Superior de Investigaciones Científicas (IHSM-UMA-CSIC), Universidad de Málaga, Málaga, Spain; Department of Microbial Ecology, Netherlands Institute of Ecology (NIOO-KNAW), Wageningen, The Netherlands; Interdisciplinary Centre of Marine and Environmental Research (CIIMAR), University of Porto, Portugal; Faculty of Sciences, University of Porto, 4150-179 Porto, Portugal; Instituto de Pesquisas de Produtos Naturais Walter Mors, Universidade Federal do Rio de Janeiro, Rio de Janeiro, RJ, 21941-599, Brazil; The Novo Nordisk Foundation Center for Biosustainability, Technical University of Denmark, Kgs. Lyngby, Denmark; Institute of Biology, Leiden University, Sylviusweg 72, 2333BE Leiden, The Netherlands; University of Strathclyde, Strathclyde Institute of Pharmacy and Biomedical Sciences, 141 Cathedral Street, Glasgow, G4 ORE UK; Translational Genome Mining for Natural Products, Interfaculty Institute of Microbiology and Infection Medicine Tübingen (IMIT), University of Tübingen, Tübingen, Germany; Interfaculty Institute for Biomedical Informatics (IBMI), University of Tübingen, Tübingen, Germany; Department of Molecular Microbiology, John Innes Centre, Norwich Research Park, Norwich, NR4 7UH, UK; Department of Embryology, Carnegie Institution for Science, 3520 San Martin Drive, Baltimore, MD 21218, USA; Department of Chemical and Pharmaceutical Biology, Groningen Research Institute of Pharmacy, University of Groningen, Antonius Deusinglaan 1, 9713 AV Groningen, The Netherlands; Institute of Molecular Bio Science, Goethe-University Frankfurt, D-60438 Frankfurt am Main, Germany; LOEWE Center for Translational Biodiversity Genomics (TBG), Senckenberganlage 25, 60325 Frankfurt am Main, Germany; Bioinformatics Group, Wageningen University, Droevendaalsesteeg, 6708 PB Wageningen, The Netherlands; Department of Biochemistry, University of Johannesburg, Auckland Park, Johannesburg 2006, South Africa; Indonesian Society of Bioinformatics And Biodiversity, Indonesia; Department of Chemistry, University of Florida Scripps Biomedical Research, 110 Scripps Way, Jupiter, FL 33458, USA; Westerdijk Fungal Biodiversity Institute, Uppsalalaan 8, 3584 CT Utrecht, The Netherlands; College of Pharmacy, Sookmyung Women's University, Seoul, South Korea; Department of Chemistry, University of Florida Scripps Biomedical Research, 110 Scripps Way, Jupiter, FL 33458, USA; Korean Lichen Research Institute, Sunchon National Universtiy, Suncheon, South Korea; Department of Chemical & Biomolecular Engineering, University of Delaware, Newark, DE 19716, USA; Department of Chemistry, The University of Hong Kong, Pokfulam Road, Hong Kong, P.R. China; Department of Biological Engineering, Massachusetts Institute of Technology, Cambridge, MA, USA; Laboratory of Microbiology, Wageningen University, Stippeneng 4, 6708WE, Wageningen, The Netherlands; Bioinformatics Group, Wageningen University, Droevendaalsesteeg, 6708 PB Wageningen, The Netherlands; Bioinformatics Group, Wageningen University, Droevendaalsesteeg, 6708 PB Wageningen, The Netherlands; Sustainable Soils and Crops, Rothamsted Research, Harpenden, Hertfordshire, UK; Instituto de Investigaciones Farmacéuticas (INIFAR), Facultad de Farmacia, Universidad de Costa Rica, San José, 11501-2060, Costa Rica; Centro de Investigaciones en Productos Naturales (CIPRONA), Universidad de Costa Rica, San José, 11501-2060, Costa Rica; Centro Nacional de Innovaciones Biotecnológicas (CENIBiot), CeNAT-CONARE, 1174-1200, San José, Costa Rica; Department of Pharmaceutical Sciences, Oregon State University, USA; Translational Genome Mining for Natural Products, Interfaculty Institute of Microbiology and Infection Medicine Tübingen (IMIT), University of Tübingen, Tübingen, Germany; Interfaculty Institute for Biomedical Informatics (IBMI), University of Tübingen, Tübingen, Germany; Bioinformatics Group, Wageningen University, Droevendaalsesteeg, 6708 PB Wageningen, The Netherlands; Interdisciplinary Centre of Marine and Environmental Research (CIIMAR), University of Porto, Portugal; Institute of Biomedical Sciences Abel Salazar (ICBAS), University of Porto, Portugal; Centre for Integrative Omics Data Science, Yenepoya (Deemed to be University), Mangalore 575018, India; Department of Environmental Microbiology, Eawag: Swiss Federal Institute for Aquatic Science and Technology, Überlandstrasse 133, CH-8600 Dübendorf, Switzerland; Institute of Biology, Leiden University, Sylviusweg 72, 2333BE Leiden, The Netherlands; Laboratorio Nacional de Genómica para la Biodiversidad-Unidad de Genómica Avanzada, Cinvestav. Km 9.6 Libramiento Norte Carretera Irapuato-León, CP 36824 Irapuato, Gto., México; School of Chemistry, University of Nottingham, University Park, Nottingham NG7 2RD, UK; Laboratory of Microbiology, Wageningen University, Stippeneng 4, 6708WE, Wageningen, The Netherlands; University of Strathclyde, Strathclyde Institute of Pharmacy and Biomedical Sciences, 141 Cathedral Street, Glasgow, G4 ORE UK; Bioinformatics Group, Wageningen University, Droevendaalsesteeg, 6708 PB Wageningen, The Netherlands; Department of Chemical and Pharmaceutical Biology, Groningen Research Institute of Pharmacy, University of Groningen, Antonius Deusinglaan 1, 9713 AV Groningen, The Netherlands; Institute of Chemical Biology, Shenzhen Bay Laboratory, Shenzhen 518132, China; DOE Joint Genome Institute, Lawrence Berkeley National Lab, Berkeley, CA, USA; Department of Microbiology, University of Szeged, Hungary; Host-Microbe Interactomics Group, Wageningen University, 6708 WD Wageningen, The Netherlands; NAICONS Srl, 20139 Milan, Italy; Bioinformatics Group, Wageningen University, Droevendaalsesteeg, 6708 PB Wageningen, The Netherlands; School of Life Sciences, The University of Warwick, Coventry CV4 7AL, UK; School of Biochemistry, University of Bristol, University Walk, Bristol BS8 1TD, UK; Department of Medicinal Chemistry, University of Utah, Salt Lake City, UT 84112, USA; Department of Chemistry and Biomolecular Sciences, University of Ottawa, Ottawa, Canada; Bioinformatics Group, Wageningen University, Droevendaalsesteeg, 6708 PB Wageningen, The Netherlands; Key laboratory of Detection for Biotoxins, Ministry of Agriculture and Rural Affairs and Oil Crops Research Institute, Chinese Academy of Agricultural Sciences, Wuhan 430061, China; Department of Chemistry and Natural Products Discovery Center, UF Scripps Biomedical Research, University of Florida, Jupiter, FL 33458, USA; SUSTech-PKU Institute of Plant and Food Science, Department of Biology, School of Life Sciences, Southern University of Science and Technology, Shenzhen, Guangdong 518055, China; Bioinformatics Group, Wageningen University, Droevendaalsesteeg, 6708 PB Wageningen, The Netherlands; Laboratory of Microbiology, Wageningen University, Stippeneng 4, 6708WE, Wageningen, The Netherlands; Westerdijk Fungal Biodiversity Institute, Uppsalalaan 8, 3584 CT Utrecht, The Netherlands; Department of Chemistry, Simon Fraser University, 8888 University Drive, Burnaby, British Columbia V5A 1S6, Canada; The Novo Nordisk Foundation Center for Biosustainability, Technical University of Denmark, Kgs. Lyngby, Denmark; Bioinformatics Group, Wageningen University, Droevendaalsesteeg, 6708 PB Wageningen, The Netherlands; Institute of Biology, Leiden University, Sylviusweg 72, 2333BE Leiden, The Netherlands

## Abstract

With an ever-increasing amount of (meta)genomic data being deposited in sequence databases, (meta)genome mining for natural product biosynthetic pathways occupies a critical role in the discovery of novel pharmaceutical drugs, crop protection agents and biomaterials. The genes that encode these pathways are often organised into biosynthetic gene clusters (BGCs). In 2015, we defined the Minimum Information about a Biosynthetic Gene cluster (MIBiG): a standardised data format that describes the minimally required information to uniquely characterise a BGC. We simultaneously constructed an accompanying online database of BGCs, which has since been widely used by the community as a reference dataset for BGCs and was expanded to 2021 entries in 2019 (MIBiG 2.0). Here, we describe MIBiG 3.0, a database update comprising large-scale validation and re-annotation of existing entries and 661 new entries. Particular attention was paid to the annotation of compound structures and biological activities, as well as protein domain selectivities. Together, these new features keep the database up-to-date, and will provide new opportunities for the scientific community to use its freely available data, e.g. for the training of new machine learning models to predict sequence-structure-function relationships for diverse natural products. MIBiG 3.0 is accessible online at https://mibig.secondarymetabolites.org/.

## Graphical Abstract

**Graphical Abstract:**
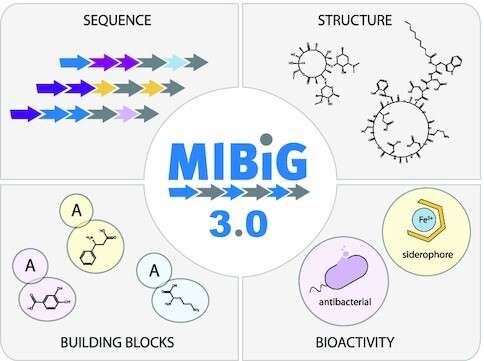
MIBiG version 3.0 provides an updated database of experimentally characterized biosynthetic gene clusters. It includes 661 new entries, as well as new structures, bioactivities and enzyme domain substrate specificities.

## INTRODUCTION

Across all kingdoms of life, organisms produce specialised metabolites: molecules that are produced by bacteria, fungi and plants to gain an advantage over their competitors in challenging environments. Specialised metabolites, also referred to as secondary metabolites or natural products, exhibit a wide variety of biological activities, including many that are useful for pharmaceutical and agricultural applications, e.g. antibiotics, anti-cancer drugs, pesticides and herbicides. The production of specialised metabolites is typically encoded by biosynthetic gene clusters (BGCs): groups of co-localised and co-regulated genes that jointly encode a biosynthetic pathway. Therefore, microbial and plant genomes can be mined for novel specialised metabolite production by detecting BGCs and predicting their encoded products and functions. Similar to how the relationship between DNA, mRNA and protein describes the flow of information in cells, we can define a ‘central dogma’ of specialised metabolism: a BGC sequence encodes a set of enzymes, which together assemble a compound structure (or a cocktail of structural analogues), which in turn dictates specialised metabolite function. Understanding how information is translated from sequence to structure to function is key to natural product discovery. To address the first stage, sequence information, various tools have been developed that automatically detect BGCs from DNA sequence, including antiSMASH and its siblings fungiSMASH and plantiSMASH (1,2), GECCO (3), DeepBGC (4), RiPPMiner (5) and PRISM 4 (6).

To facilitate dereplication and comparative analysis of predicted BGCs with known BGCs, and to characterise the interplay between sequence, structure and function, standardised data annotation and storage are essential. To this purpose, we developed the Minimum Information about a Biosynthetic Gene cluster (MIBiG) standard and built a database which contains standardised entries for experimentally validated BGCs of known function (7,8). Each entry minimally contains information about the nucleotide entry and coordinates of the genomic locus involved, the producing organism's taxonomy, biosynthetic class, name of the produced compound(s), and literature reference(s). There are also various optional fields for non-minimal entries, including fields for gene function, product structure and bioactivity, crosslinks to chemical structure databases such as NP Atlas (9) and PubChem (10), and monomer identity. With MIBiG 2.0 containing over 2000 entries, the database has become an important reference for many researchers that mine genomes for natural products. For example, it has been used to estimate the potential for biosynthetic novelty in large-scale microbiome studies (11,12), to identify conserved amino acids playing key roles in catalytic activities across enzyme families (13), to help guide natural product discovery efforts towards high-potential taxa (14), and to train machine-learning algorithms for natural product activity prediction (15).

**Figure 1.**
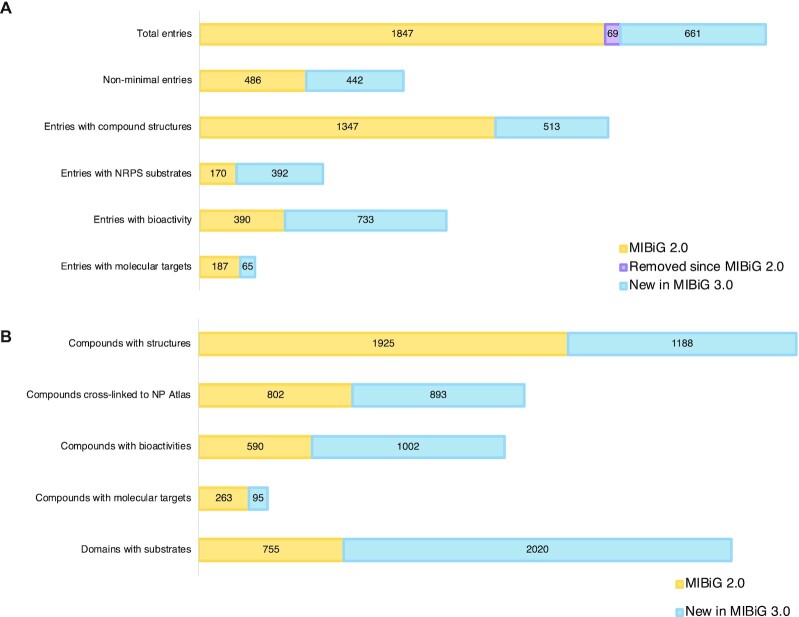
Overview of MIBiG 3.0. (**A**) Added, removed and updated entries since MIBiG 2.0. (**B**) Improvements in the annotation of compounds, bioactivities, molecular targets and NRPS domain substrates.

Here, we present MIBiG 3.0: an update designed to increase the number of non-minimal entries in our database and adding new data entries through a large-scale community annotation effort. We focused on three features: the characterisation and cross-linking of 1188 chemical structures, the annotation of 1002 bioactivities of BGC products, and the validation and annotation of 2020 protein domain substrates of nonribosomal peptide synthetases (NRPSs). In addition, we added 661 novel BGCs to the MIBiG database which were published since the last database update and removed 69 duplicate and low-quality entries (Figure 1). Together, these additions keep the database current, and provide unique opportunities for exploring complex sequence-structure-function relationships in diverse natural product domains.

## METHODS AND IMPLEMENTATION

### Manual curation through crowdsourcing and mass online ‘annotathons’

As authors themselves typically have the best understanding of the BGC they have studied, we greatly encourage natural product researchers to submit their BGCs to MIBiG during the process of publishing their work. To this purpose, MIBiG supplies an online form through which researchers can request a unique MIBiG identifier and submit their experimentally verified BGCs, pre- or post-publication. Since MIBiG version 2.0, this has yielded 97 manually submitted, high-quality entries which have now been incorporated into MIBiG 3.0. Still, there are far more published BGCs that are not manually submitted to MIBiG.

With an increasing number of papers describing novel BGCs being published every year, manually annotating, validating and adding BGCs to MIBiG has become a mammoth task. Therefore, we took to social media to gauge the community's interest in participating in an online annotation event. We received many positive responses, with 86 people from four different continents volunteering to participate in our MIBiG ‘annotathons’. We organised eight three-hour online sessions, accommodating different time-zones, with various breakout rooms dedicated to specific annotation tasks: annotating new clusters, annotating and cross-linking compound structures, annotating compound bioactivities, and assigning substrate selectivities to NRPS protein domains. We prepared multiple instruction videos and assigned an expert to each of the breakout rooms who could be directly approached with questions from annotators to ensure that annotation quality was consistent. In addition, one of our annotators at the CINVESTAV research institute mobilised fourteen MSc Integrative Biology students of their 2021 Bacterial Genomics class to annotate compound bioactivities under supervision. Finally, we resolved 125 database issues that were raised by users on our GitHub page, redefining BGC boundaries, correcting biosynthetic classes, adding and removing literature references, fixing compound structures, and removing duplicate entries.

### Annotating and cross-linking compound structures

Since version 2.0, compound structures in MIBiG have been cross-linked to the NP Atlas database: a database containing structures of natural products isolated from bacteria and fungi. During the preparations for version 3.0, we collaborated with the NP Atlas team to (i) add structures for compounds in SMILES format (16), including stereochemical information where possible and (ii) cross-link them to five databases of chemical structures: NP Atlas (9), PubChem, ChemSpider (17), LOTUS (18), and ChEMBL (19). If compound entries were found in multiple databases, SMILES strings from NP Atlas were prioritised. SMILES strings were also collected for existing entries that were already cross-linked to a database but did not report a SMILES string. Correctness of SMILES syntax was validated with PIKAChU (20).

### Annotating compound bioactivities

To improve MIBiG as a resource for machine learning models predicting sequence-structure-function relationships, we added bioactivity data for 1002 compounds and chemical target data for 95 compounds. 708 of these annotations were transferred from the dataset assembled by Walker and Clardy, who designed a machine learning model to predict BGC function from sequence (15). To accommodate consistent annotations, we assigned all existing and novel bioactivities to 68 standardised functional categories (Supplementary Table S1).

### Annotating NRPS protein domains

To concretise the relationship between NRPS sequence and the structure of its produced nonribosomal peptide (NRP), we annotated and validated the substrate selectivities of 2775 NRPS adenylation (A) domains. A-domains dictate which monomers (predominantly amino acids) are incorporated into (hybrid) NRP scaffolds. Substrate annotation can be performed at different levels: we can define the pre-tailored substrate precursor (e.g. l-aspartic acid); the substrate as recognised by the A-domain (e.g. (3*R*)-3-hydroxy-l-aspartic acid); or the post-tailored integrated monomer that ends up in the final NRP scaffold (e.g. (3*R*)-3-hydroxy-d-aspartic acid). We chose to annotate the substrates as recognised by the A-domain, as this best reflects the biological relationship between A-domain and incorporated monomer. In addition to substrate identity, we also recorded evidence for substrate selectivity in the form of an evidence code and literature references. To this purpose, we added 13 evidence codes to the JSON schema which is used to standardise MIBiG entries (Table 1).

**Table 1.** Evidence codes for adenylation domain substrate annotations

Evidence code	Accepted as standalone evidence	New in MIBiG 3.0
Activity assay	X	
ACVS assay	X	X
ATP-PPi exchange assay	X	X
Enzyme-coupled assay	X	X
Feeding study	X	
Heterologous expression	X	X
Homology		X
HPLC	X	X
*In-vitro* experiments	X	X
Knock-out studies	X	X
Mass spectrometry	X	X
NMR	X	X
Radio labelling	X	X
Sequence-based prediction		
Steady-state kinetics	X	X
Structure-based inference	X	
X-ray crystallography	X	X

As indicated, some evidence codes are only accepted as evidence for substrate specificity when combined with a second evidence code that provides further support for a data entry. Thirteen evidence codes were newly introduced in MIBiG 3.0. ACVS assay: δ-(l-*R*-aminoadipyl)-l-cysteinyl-d-valine synthetase assay, specific for measuring penicillin production. HPLC: high-performance liquid chromatography. NMR: nuclear magnetic resonance.

**Figure 2.**
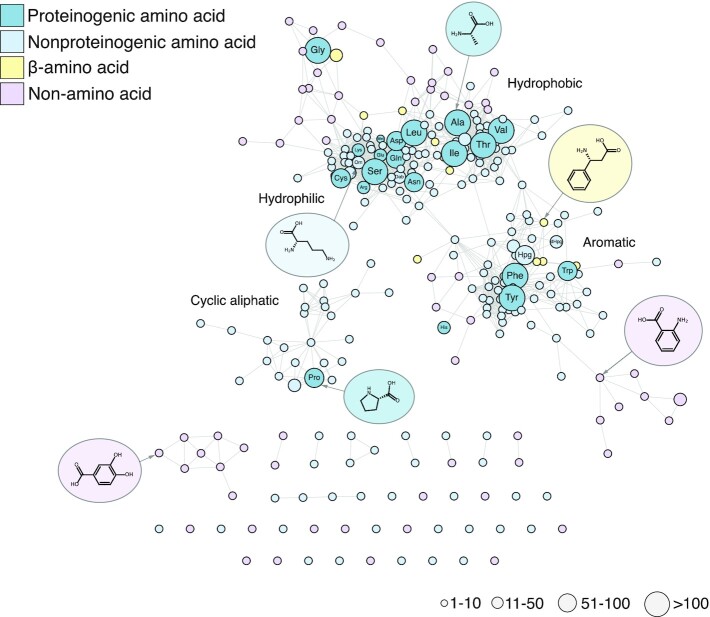
Similarity network of annotated NRPS substrates. Each node represents one of 274 unique NRPS substrate structures in MIBiG 3.0. Colours indicate substrate categories, and node size correlates with the number of annotations for that substrate in the MIBiG database. Substrates were clustered based on Tanimoto similarity of ECFP-4 molecular fingerprints (25) (edge cut-off = 0.46).

After community annotation, substrate naming was homogenised and each stereochemically ambiguous substrate was manually curated by an expert. Where stereochemistry could be inferred from structure, this is reflected in the substrate name for each stereocenter. Exceptions are amino acid names, which are assumed to be in their l-configuration. To avoid any ambiguity in substrate naming, we also linked each of our 274 unique substrate names to an isomeric SMILES string representing the substrate structure (Figure 2; Supplementary Table S2). SMILES validation and deduplication were handled using PIKAChU (20).

## RESULTS AND DISCUSSION

### Taking the ‘minimal’ out of MIBiG

While MIBiG 2.0 serves an important role in the community as a reference database to quickly identify whether a BGC is similar to any known BGCs, its utility as a resource for exploring sequence-structure-function relationships could be improved. This can mainly be explained by the high number of minimal entries in the database: entries that only contain sequence and compound information that could be augmented by adding further standardised annotations. For MIBiG 3.0, we aimed to promote as many existing and novel entries as possible to non-minimal entries by annotating compound structures (1188), bioactivities (1002) and NRPS substrates (2020). In total, we added 661 novel BGCs and 4871 separate data entries to our database, increasing our number of non-minimal entries from 486 to 928 (Figure 1, Supplementary Figure S1). MIBiG 3.0 now contains 2502 entries, spanning 16 phyla across 5 kingdoms of life (Table 2).

**Table 2.** Entries in MIBiG 3.0 by phylum

Kingdom	Phylum	Number of BGCs in MIBiG 3.0
Bacteria	Actinobacteria	1042
	Proteobacteria	527
	Firmicutes	229
	Cyanobacteria	139
	Bacteroidetes	17
	Candidatus tectomicrobia	6
	Chloroflexi	4
	Verrucomicrobia	3
	Planctomycetes	2
	Kiritimatiellaeota	1
	Unknown	41
Fungi	Ascomycota	415
	Basidiomycota	23
	Unknown	3
Plantae	Streptophyta	43
	Rhodophyta	2
Archaea	Euryarchaeota	3
Chromista	Bacillariophyta	1
	Dinophyceae	1

### Streamlining research into the central dogma of specialised metabolism

With 905 NRPS and modular Type I PKS BGCs in MIBiG 3.0, modular BGCs constitute a substantial part of our database. Modular systems are characterised by enzyme complexes comprising repeating domain architectures, which collectively assemble a natural product scaffold. When the substrate selectivities of the recognition domains are known (acyltransferase (AT) domains for PKS and A-domains for NRPS), these consistent architectures make it possible to predict the structure of chemical scaffolds with reasonable accuracy. Most AT domains in PKS systems recognise one of two substrates, malonyl-CoA or methylmalonyl-CoA, and excellent bioinformatics tools exist to distinguish between the two (21). However, for A-domains in NRPS systems, which recognise over 500 known substrates (22), substrate prediction is a greater challenge, which will require substantially more data to obtain models of comparably predictive power. Therefore, we decided to make the annotation of the substrate selectivity of NRPS A-domains a major focus of MIBiG 3.0. MIBiG 3.0 now contains annotations for 2775 A-domains (compared to 755 annotations in MIBiG 2.0; Figure 1B), covering 274 unique substrates which are identified by stereochemically curated isomeric SMILES strings (Figure 2; Supplementary Table S2). This makes MIBiG the largest resource for A-domain substrate data, containing 3–4 times as many labelled data points as the training sets used for the A-domain selectivity predictors SANDPUMA (23) and NRPSPredictor2 (24). We hope that eventually this dataset will be leveraged to train an improved A-domain substrate predictor, which can in turn be integrated into tools like antiSMASH to improve NRP scaffold structure prediction.

Since version 2.0, we have added structural identifiers of 1188 compounds to our database in SMILES format (16), increasing the number of BGCs with structural data from 1347 to 1860 (Figure 1). By pulling SMILES strings directly from cross-linked databases where possible, we avoid conflicts caused by versioning and SMILES formatting. Additionally, we linked 1002 additional compounds to 51 unique bioactivities, creating opportunities for computationally predicting compound bioactivity from structure. For a further 95 compounds, we were also able to annotate their molecular targets (Figure 1B).

By centering MIBiG 3.0 around the annotation of substrate building blocks, compound structures, and bioactivities, we aspired to streamline future research into all aspects of sequence-structure-function relationships that lie at the heart of natural product research. All data can be easily downloaded and parsed in bulk from our database in JSON and GenBank format or accessed on an entry-by-entry basis through our searchable online repository. As such, we hope that MIBiG 3.0 will prove an important resource for future machine learning endeavours that aim to decode the central dogma of specialised metabolism.

## DATA AVAILABILITY

The MIBiG Repository is available at https://mibig.secondarymetabolites.org/. There is no access restriction for academic or commercial use of the repository and its data. The source code components, JSON-formatted data standard, and SQL schema for the MIBiG Repository are available on GitHub (https://github.com/mibig-secmet) under an OSI-approved Open Source licence.

## Supplementary Material

gkac1049_Supplemental_Files
