# Supplementary material for: MIBiG 3.0: a community-driven effort to annotate experimentally validated biosynthetic gene clusters
Source: Nucleic Acids Res. 2022 Nov 18;51(D1):D603–10. doi: 10.1093/nar/gkac1049 (PMC9825592; doi:10.1093/nar/gkac1049)
Supplement: gkac1049_Supplemental_Files [file gkac1049_supplemental_files.zip › Figure_S1.pdf]

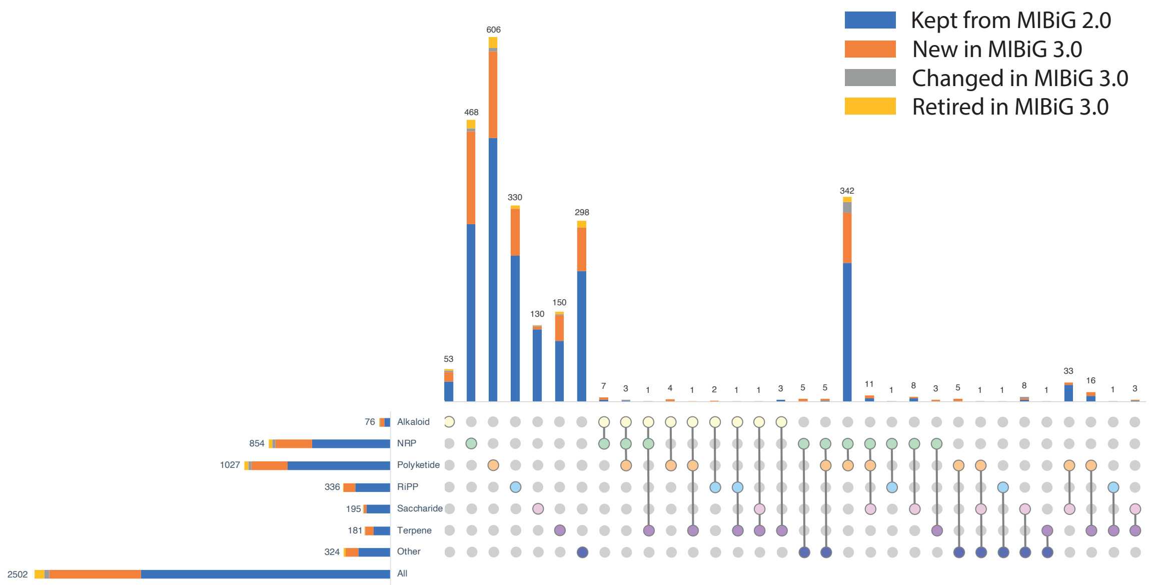

Supplementary Figure 1. MIBiG 3.0 BGC diversity, categorised by biosynthetic class. Horizontal bar plots show total occurrences of BGC biosynthetic (sub)classes in MIBiG 2.0 and 3.0; vertical bars show counts of BGCs of a certain (hybrid) biosynthetic class. Old entries, new entries, retired entries, and entries whose biosynthetic class has changed between versions are indicated. Numbers to the left of bars and above bars represent the total number of subclass occurrences/entries in MIBiG 3.0, not counting retired entries.
